# *Linosporopsis*, a new leaf-inhabiting scolecosporous genus in Xylariaceae

**DOI:** 10.1007/s11557-020-01559-7

**Published:** 2020-02-08

**Authors:** Hermann Voglmayr, Ludwig Beenken

**Affiliations:** 1 Institute of Forest Entomology, Forest Pathology and Forest Protection, Dept. of Forest and Soil Sciences, BOKU-University of Natural Resources and Life Sciences, Franz Schwackhöfer Haus, Peter-Jordan-Straße 82/I, 1190 Vienna, Austria; 2 Division of Systematic and Evolutionary Botany, Department of Botany and Biodiversity Research, University of Vienna, Rennweg 14, 1030 Wien, Austria; 3 Eidgenössische Forschungsanstalt WSL, Zürcherstrasse 111, 8903 Birmensdorf, Switzerland

**Keywords:** Ascomycota, Diaporthales, Leaf endophytes, *Linospora*, Molecular phylogeny, Systematics, Xylariales, 4 new combinations, 1 new name

## Abstract

Based on molecular phylogenetic and morphological evidence, the new genus *Linosporopsis* (Xylariales) is established for several species previously classified within *Linospora* (Diaporthales). Fresh collections of *Linospora ischnotheca* from dead overwintered leaves of *Fagus sylvatica* and of *L. ochracea* from dead overwintered leaves of *Malus domestica*, *Pyrus communis*, and *Sorbus intermedia* were isolated in pure culture, and molecular phylogenetic analyses of a multi-locus matrix of partial nuITS-LSU rDNA, *RPB2* and *TUB2* sequences as well as morphological investigations revealed that both species are unrelated to the diaporthalean genus *Linospora*, but belong to Xylariaceae sensu stricto. The new combinations *Linosporopsis ischnotheca* and *L. ochracea* are proposed, the species are described and illustrated, and their basionyms lecto- and epitypified. *Linospora faginea* is synonymized with *L. ischnothe*ca. Based on similar morphology and ecology, *Linospora carpini* and *Linospora magnagutiana* from dead leaves of *Carpinus betulus* and *Sorbus torminalis*, respectively, are also combined in *Linosporopsis*. The four accepted species of *Linosporopsis* are illustrated, a key to species is provided and their ecology is discussed.

## Introduction

The genus *Linospora* was established by Fuckel (1870) for five species growing on dead leaves of Salicaeae. He did not designate a generic type, but Clements and Shear (1931) selected *Linospora capreae*, which grows on *Salix caprea*, as lectotype. The genus is characterized by long, filiform ascospores arranged in a single fascicle within the ascus, and by reduced black stromata embedded in dead leaf tissue containing usually one (in *L. ceuthocarpa* up to six) perithecia with laterally inserted ostioles. The black stromata appear in spring and are noticeable as black dots of ca. 0.5–1 mm diam on both sides of the dead, usually bleached leaves. The characteristics of ascomata and asci are clearly diaporthalean, and its classification within Gnomoniaceae (Monod 1983; Barr 1990) has also been corroborated by molecular phylogenetic analyses (Mejía et al. 2008). So far, the about eight accepted species of *Linospora* inhabit leaves of *Salix* or *Populus* spp. (Salicaceae), but morphological evidence suggests the presence of additional undescribed species on Salicaceae (Monod 1983).

Soon after its description, additional species with long filiform ascospores and black ascomata or stromata embedded in leaf tissues were added to *Linospora*. However, critical morphological re-investigations by Monod (1983) revealed that many of these are not diaporthalean and therefore unrelated to the generic type. Five of them, *L. carpini* from leaves of *Carpinus betulus*; *L. faginea*, and *L. ischnotheca* from leaves of *Fagus sylvatica*; *L. magnagutiana* from leaves of *Sorbus torminalis* and *L. ochracea* from leaves of various other rosaceous hosts from subtribe Pyrinae, were considered to be synonymous and to belong to the genus *Ophiodothella* (Phyllachoraceae), but Monod (1983) neither provided a detailed reasoning nor proposed a formal combination. Thus, in the lack of additional detailed studies, the nomenclature, systematic affiliation and taxonomic status of these five species remained unresolved.

Recent fresh collections of *L. ischnotheca* and *L. ochracea* provided the opportunity to study their morphology in detail and to isolate them in pure culture for sequencing. Molecular phylogenetic analyses of a multi-locus matrix of nuITS-LSU rDNA, *RPB2* and *TUB2* sequences and morphological studies including type material enabled us to resolve their systematic affiliation, to evaluate their species status and taxonomy, and to propose a revised classification, the results of which we report here.

## Materials and methods

### Sample sources

All isolates included in this study originated from ascospores of freshly collected specimens. Details of the strains including NCBI GenBank accession numbers of gene sequences used to compute the phylogenetic trees are listed in Table 1. Strain acronyms other than those of official culture collections are used here primarily as strain identifiers throughout the work. Representative isolates have been deposited at the Westerdijk Fungal Biodiversity Centre (CBS-KNAW), Utrecht, The Netherlands. Details of the specimens used for morphological investigations are listed in the Taxonomy section under the respective descriptions. Herbarium acronyms are according to Thiers (2019), and citation of exsiccata follows Triebel and Scholz (2019). Specimens have been deposited in the Fungaria of the Department of Botany and Biodiversity Research, University of Vienna (WU) and of the Eidgenössische Technische Hochschule Zürich (ZT).

**Table 1:** Isolates and accession numbers used in the phylogenetic analyses. Isolates/sequences in bold were isolated/sequenced in the present study

Species	Specimen or strain number^a^	Origin	Status^b^	GenBank accession numbers^c^				References
				ITS	LSU	*RPB2*	*TUB2*	
*Amphirosellinia fushanensis*	HAST 91111209	Taiwan	HT	GU339496	N/A	GQ848339	GQ495950	Hsieh et al. (2010)
*Amphirosellinia nigrospora*	HAST 91092308	Taiwan	HT	GU322457	N/A	GQ848340	GQ495951	Hsieh et al. (2010)
*Annulohypoxylon annulatum*	CBS 140775	Texas	ET	KY610418	KY610418	KY624263	KX376353	Kuhnert et al. (2017), Wendt et al. (2018)
*Annulohypoxylon atroroseum*	ATCC 76081	Thailand		AJ390397	KY610422	KY624233	DQ840083	Kuhnert et al. (2014), Wendt et al. (2018)
*Annulohypoxylon michelianum*	CBS 119993	Spain		KX376320	KY610423	KY624234	KX271239	Kuhnert et al. (2014), Wendt et al. (2018)
*Annulohypoxylon moriforme*	CBS 123579	Martinique		KX376321	KY610425	KY624289	KX271261	Kuhnert et al. (2017), Wendt et al. (2018)
*Annulohypoxylon nitens*	MFLUCC 12–0823	Thailand		KJ934991	KJ934992	KJ934994	KJ934993	Daranagama et al. (2015)
*Annulohypoxylon stygium*	MUCL 54601	French Guiana		KY610409	KY610475	KY624292	KX271263	Wendt et al. (2018)
*Annulohypoxylon truncatum*	CBS 140778	Texas	ET	KY610419	KY610419	KY624277	KX376352	Kuhnert et al. (2017), Wendt et al. (2018)
*Anthostomelloides krabiensis*	MFLUCC 15–0678	Thailand	HT	KX305927	KX305928	KX305929	N/A	Tibpromma et al. (2017)
*Astrocystis concavispora*	MFLUCC 14–0174	Italy		KP297404	KP340545	KP340532	KP406615	Daranagama et al. (2015)
*Barrmaelia macrospora*	CBS 142768	Austria	ET	KC774566	KC774566	MF488995	MF489014	Jaklitsch et al. 2014, Voglmayr et al. (2018)
*Barrmaelia moravica*	CBS 142769	Austria	ET	MF488987	MF488987	MF488996	MF489015	Voglmayr et al. (2018)
*Barrmaelia oxyacanthae*	CBS 142770	Austria		MF488988	MF488988	MF488997	MF489016	Voglmayr et al. (2018)
*Barrmaelia rappazii*	CBS 142771	Norway	HT	MF488989	MF488989	MF488998	MF489017	Voglmayr et al. (2018)
*Barrmaelia rhamnicola*	CBS 142772	France	ET	MF488990	MF488990	MF488999	MF489018	Voglmayr et al. (2018)
*Biscogniauxia arima*	WSP 122	Mexico	IT	EF026150	N/A	GQ304736	AY951672	Hsieh et al. (2005, 2010)
*Biscogniauxia atropunctata*	Y.M.J. 128	USA		JX507799	N/A	JX507778	AY951673	Hsieh et al. (2005), Mirabolfathy et al. (2013)
*Biscogniauxia marginata*	MFLUCC 12–0740	France		KJ958407	KJ958408	KJ958409	KJ958406	Daranagama et al. (2015)
*Biscogniauxia nummularia*	MUCL 51395	France	ET	KY610382	KY610427	KY624236	KX271241	Wendt et al. (2018)
*Biscogniauxia repanda*	ATCC 62606	USA		KY610383	KY610428	KY624237	KX271242	Wendt et al. (2018)
*Camillea obularia*	ATCC 28093	Puerto Rico		KY610384	KY610429	KY624238	KX271243	Wendt et al. (2018)
*Camillea tinctor*	Y.M.J. 363	Martinique		JX507806	N/A	JX507790	JX507795	Mirabolfathy et al. (2013)
*Clypeosphaeria mamillana*	CBS 140735	France	ET	KT949897	KT949897	MF489001	N/A	Jaklitsch et al. 2016, Voglmayr et al. (2018)
*Collodiscula bambusae*	GZU H0102	China		KP054279	KP054280	KP276675	KP276674	Li et al. (2015)
*Collodiscula fangjingshanensis*	GZU H0109	China	HT	KR002590	KR002591	KR002592	KR002589	Li et al. (2015)
*Collodiscula japonica*	CBS 124266	China		JF440974	JF440974	KY624273	KY624316	Jaklitsch and Voglmayr (2012), Wendt et al. (2018)
*Creosphaeria sassafras*	STMA 14087	Argentina		KY610411	KY610468	KY624265	KX271258	Wendt et al. (2018)
*Daldinia andina*	CBS 114736	Ecuador	HT	AM749918	KY610430	KY624239	KC977259	Bitzer et al. (2008), Kuhnert et al. (2014), Wendt et al. (2018)
*Daldinia bambusicola*	CBS 122872	Thailand	HT	KY610385	KY610431	KY624241	AY951688	Hsieh et al. (2005), Wendt et al. (2018)
*Daldinia caldariorum*	MUCL 49211	France		AM749934	KY610433	KY624242	KC977282	Bitzer et al. (2008), Kuhnert et al. (2014), Wendt et al. (2018)
*Daldinia concentrica*	CBS 113277	Germany		AY616683	KY610434	KY624243	KC977274	Triebel et al. (2005), Kuhnert et al. (2014), Wendt et al. (2018)
*Daldinia dennisii*	CBS 114741	Australia	HT	JX658477	KY610435	KY624244	KC977262	Stadler et al. (2014), Kuhnert et al. (2014), Wendt et al. (2018)
*Daldinia eschscholtzii*	MUCL 45435	Benin		JX658484	KY610437	KY624246	KC977266	Stadler et al. (2014), Kuhnert et al. (2014), Wendt et al. (2018)
*Daldinia loculatoides*	CBS 113279	UK	ET	AF176982	KY610438	KY624247	KX271246	Johannesson et al. (2000), Wendt et al. (2018)
*Daldinia macaronesica*	CBS 113040	Spain	PT	KY610398	KY610477	KY624294	KX271266	Wendt et al. (2018)
*Daldinia petriniae*	MUCL 49214	Austria	ET	AM749937	KY610439	KY624248	KC977261	Bitzer et al. (2008), Kuhnert et al. (2014), Wendt et al. (2018)
*Daldinia placentiformis*	MUCL 47603	Mexico		AM749921	KY610440	KY624249	KC977278	Bitzer et al. (2008), Kuhnert et al. (2014), Wendt et al. (2018)
*Daldinia pyrenaica*	MUCL 53969	France		KY610413	KY610413	KY624274	KY624312	Wendt et al. (2018)
*Daldinia steglichii*	MUCL 43512	Papua New Guinea	PT	KY610399	KY610479	KY624250	KX271269	Wendt et al. (2018)
*Daldinia theissenii*	CBS 113044	Argentina	PT	KY610388	KY610441	KY624251	KX271247	Wendt et al. (2018)
*Daldinia vernicosa*	CBS 119316	Germany	ET	KY610395	KY610442	KY624252	KC977260	Kuhnert et al. (2014), Wendt et al. (2018)
*Diatrype disciformis*	CBS 197.49	Netherlands		N/A	DQ470964	DQ470915	N/A	Zhang et al. (2006)
*Entoleuca mammata*	J.D.R. 100	France		GU300072	N/A	GQ844782	GQ470230	Hsieh et al. (2010)
*Entonaema liquescens*	ATCC 46302	USA		KY610389	KY610443	KY624253	KX271248	Wendt et al. (2018)
*Entosordaria perfidiosa*	CBS 142773	Austria	ET	MF488993	MF488993	MF489003	MF489021	Voglmayr et al. (2018)
*Entosordaria quercina*	CBS 142774	Greece	HT	MF488994	MF488994	MF489004	MF489022	Voglmayr et al. (2018)
*Euepixylon sphaeriostomum*	J.D.R. 261	USA		GU292821	N/A	GQ844774	GQ470224	Hsieh et al. (2010)
*Eutypa lata*	UCR-EL1	USA		JGI	JGI	JGI	JGI	
*Gnomonia gnomon*	CBS 199.53	Italy		AY818956	AF408361	EU219295	EU219148	Castlebury et al. (2002), Sogonov et al. (2005, 2008)
*Graphostroma platystomum*	CBS 270.87	France		JX658535	DQ836906	KY624296	HG934108	Zhang et al. (2006), Stadler et al. (2014), Koukol et al. (2015), Wendt et al. (2018)
*Hypocreodendron sanguineum*	J.D.R. 169	Mexico		GU322433	N/A	GQ844819	GQ487710	Hsieh et al. (2010)
*Hypomontagnella monticulosa*	MUCL 54604	French Guiana	ET	KY610404	KY610487	KY624305	KX271273	Wendt et al. (2018)
*Hypomontagnella submonticulosa*	CBS 115280	France		KC968923	KY610457	KY624226	KC977267	Kuhnert et al. (2014), Wendt et al. (2018)
*Hypoxylon carneum*	MUCL 54177	France		KY610400	KY610480	KY624297	KX271270	Wendt et al. (2018)
*Hypoxylon cercidicola*	CBS 119009	France		KC968908	KY610444	KY624254	KC977263	Kuhnert et al. (2014), Wendt et al. (2018)
*Hypoxylon crocopeplum*	CBS 119004	France		KC968907	KY610445	KY624255	KC977268	Kuhnert et al. (2014), Wendt et al. (2018)
*Hypoxylon fendleri*	MUCL 54792	French Guiana		KF234421	KY610481	KY624298	KF300547	Kuhnert et al. (2014), Wendt et al. (2018)
*Hypoxylon fragiforme*	MUCL 51264	Germany	ET	KC477229	KM186295	KM186296	KX271282	Stadler et al. (2013), Daranagama et al. (2015), Wendt et al. (2018)
*Hypoxylon fuscum*	CBS 113049	France	ET	KY610401	KY610482	KY624299	KX271271	Wendt et al. (2018)
*Hypoxylon griseobrunneum*	CBS 331.73	India	HT	KY610402	KY610483	KY624300	KC977303	Kuhnert et al. (2014), Wendt et al. (2018)
*Hypoxylon haematostroma*	MUCL 53301	Martinique	ET	KC968911	KY610484	KY624301	KC977291	Kuhnert et al. (2014), Wendt et al. (2018)
*Hypoxylon howeanum*	MUCL 47599	Germany		AM749928	KY610448	KY624258	KC977277	Bitzer et al. (2008), Kuhnert et al. (2014), Wendt et al. (2018)
*Hypoxylon hypomiltum*	MUCL 51845	Guadeloupe		KY610403	KY610449	KY624302	KX271249	Wendt et al. (2018)
*Hypoxylon investiens*	CBS 118183	Malaysia		KC968925	KY610450	KY624259	KC977270	Kuhnert et al. (2014), Wendt et al. (2018)
*Hypoxylon lateripigmentum*	MUCL 53304	Martinique	HT	KC968933	KY610486	KY624304	KC977290	Kuhnert et al. (2014), Wendt et al. (2018)
*Hypoxylon lenormandii*	CBS 119003	Ecuador		KC968943	KY610452	KY624261	KC977273	Kuhnert et al. (2014), Wendt et al. (2018)
*Hypoxylon musceum*	MUCL 53765	Guadeloupe		KC968926	KY610488	KY624306	KC977280	Kuhnert et al. (2014), Wendt et al. (2018)
*Hypoxylon ochraceum*	MUCL 54625	Martinique	ET	KC968937	N/A	KY624271	KC977300	Kuhnert et al. (2014), Wendt et al. (2018)
*Hypoxylon papillatum*	ATCC 58729	USA	HT	KC968919	KY610454	KY624223	KC977258	Kuhnert et al. (2014), Wendt et al. (2018)
*Hypoxylon perforatum*	CBS 115281	France		KY610391	KY610455	KY624224	KX271250	Wendt et al. (2018)
*Hypoxylon petriniae*	CBS 114746	France	HT	KY610405	KY610491	KY624279	KX271274	Kuhnert et al. (2014), Wendt et al. (2018)
*Hypoxylon pilgerianum*	STMA 13455	Martinique		KY610412	KY610412	KY624308	KY624315	Wendt et al. (2018)
*Hypoxylon porphyreum*	CBS 119022	France		KC968921	KY610456	KY624225	KC977264	Kuhnert et al. (2014), Wendt et al. (2018)
*Hypoxylon pulicicidum*	CBS 122622	Martinique	HT	JX183075	KY610492	KY624280	JX183072	Bills et al. (2012), Wendt et al. (2018)
*Hypoxylon rickii*	MUCL 53309	Martinique	ET	KC968932	KY610416	KY624281	KC977288	Kuhnert et al. (2014), Wendt et al. (2018)
*Hypoxylon rubiginosum*	MUCL 52887	Germany	ET	KC477232	KY610469	KY624266	KY624311	Stadler et al. (2013), Wendt et al. (2018)
*Hypoxylon samuelsii*	MUCL 51843	Guadeloupe	ET	KC968916	KY610466	KY624269	KC977286	Kuhnert et al. (2014), Wendt et al. (2018)
*Hypoxylon ticinense*	CBS 115271	France		JQ009317	KY610471	KY624272	AY951757	Hsieh et al. (2005), Wendt et al. (2018)
*Hypoxylon trugodes*	MUCL 54794	Sri Lanka	ET	KF234422	KY610493	KY624282	KF300548	Kuhnert et al. (2014), Wendt et al. (2018)
*Hypoxylon vogesiacum*	CBS 115273	France		KC968920	KY610417	KY624283	KX271275	Kuhnert et al. (2014), Kuhnert et al. (2017), Wendt et al. (2018)
*Jackrogersella cohaerens*	CBS 119126	Germany		KY610396	KY610497	KY624270	KY624314	Wendt et al. (2018)
*Jackrogersella minutella*	CBS 119015	Portugal		KY610381	KY610424	KY624235	KX271240	Kuhnert et al. (2017), Wendt et al. (2018)
*Jackrogersella multiformis*	CBS 119016	Germany	ET	KC477234	KY610473	KY624290	KX271262	Kuhnert et al. (2014), Kuhnert et al. (2017), Wendt et al. (2018)
*Juglanconis juglandina*	CBS 133343	Austria		KY427149	KY427149	KY427199	KY427234	Voglmayr et al., (2017)
*Kretzschmaria deusta*	CBS 163.93	Germany		KC477237	KY610458	KY624227	KX271251	Stadler et al. (2013), Wendt et al. (2018)
*Linospora capreae*	CBS 372.69	Netherlands		EU199194	EU255199	EU199152	EU219232	Mejía et al. (2008)
*Linosporopsis ischnotheca*	**LIF1 = CBS 145761**	Switzerland	**ET**	**MN818952**	**MN818952**	**MN820708**	**MN820715**	This study
*Linosporopsis ischnotheca*	**LIF2**	Switzerland		**MN818953**	**MN818953**	**MN820709**	**MN820716**	This study
*Linosporopsis ischnotheca*	**LIF3**	Spain		**MN818954**	**MN818954**	**MN820710**	**MN820717**	This study
*Linosporopsis ochracea*	**LIO = CBS 145760**	Switzerland		**MN818955**	**MN818955**	**MN820711**	**MN820718**	This study
*Linosporopsis ochracea*	**LIO1**	Austria		**MN818956**	**MN818956**	**MN820712**	**MN820719**	This study
*Linosporopsis ochracea*	**LIO2**	Germany		**MN818957**	**MN818957**	**MN820713**	**MN820720**	This study
*Linosporopsis ochracea*	**LIO3 = CBS 145999**	Germany	**ET**	**MN818958**	**MN818958**	**MN820714**	**MN820721**	This study
*Lopadostoma dryophilum*	CBS 133213	Austria	ET	KC774570	KC774570	KC774526	MF489023	Jaklitsch et al. 2014, Voglmayr et al. (2018)
*Lopadostoma turgidum*	CBS 133207	Austria	ET	KC774618	KC774618	KC774563	MF489024	Jaklitsch et al. 2014, Voglmayr et al. (2018)
*Melanconis stilbostoma*	D143	Poland		KY427156	KY427156	KY427206	KY427241	Voglmayr et al., (2017)
*Nemania abortiva*	BISH 467	USA	HT	GU292816	N/A	GQ844768	GQ470219	Hsieh et al. (2010)
*Nemania beaumontii*	HAST 405	Martinique		GU292819	N/A	GQ844772	GQ470222	Hsieh et al. (2010)
*Nemania bipapillata*	HAST 90080610	Taiwan		GU292818	N/A	GQ844771	GQ470221	Hsieh et al. (2010)
*Nemania maritima*	HAST 89120401	Taiwan	ET	N/A	N/A	GQ844775	GQ470225	Hsieh et al. (2010)
*Nemania maritima*	STMA 04019 = J.F. 03075	France		KY610414	KY610414	N/A	N/A	Wendt et al. (2018)
*Nemania primolutea*	HAST 91102001	Taiwan	HT	EF026121	N/A	GQ844767	EF025607	Hsieh et al. (2010)
*Obolarina dryophila*	MUCL 49882	France		GQ428316	GQ428316	KY624284	GQ428322	Pažoutová et al. (2010), Wendt et al. (2018)
*Podosordaria mexicana*	WSP 176	Mexico		GU324762	N/A	GQ853039	GQ844840	Hsieh et al. (2010)
*Podosordaria muli*	WSP 167	Mexico	HT	GU324761	N/A	GQ853038	GQ844839	Hsieh et al. (2010)
*Poronia pileiformis*	WSP 88113001	Taiwan	ET	GU324760	N/A	GQ853037	GQ502720	Hsieh et al. (2010)
*Poronia punctata*	CBS 656.78	Australia	HT	KT281904	KY610496	KY624278	KX271281	Senanayake et al. (2015), Wendt et al. (2018)
*Pyrenopolyporus hunteri*	MUCL 52673	Ivory Coast	ET	KY610421	KY610472	KY624309	KU159530	Kuhnert et al. (2017), Wendt et al. (2018)
*Pyrenopolyporus laminosus*	MUCL 53305	Martinique	HT	KC968934	KY610485	KY624303	KC977292	Kuhnert et al. (2014), Wendt et al. (2018)
*Pyrenopolyporus nicaraguensis*	CBS 117739	Burkina Faso		AM749922	KY610489	KY624307	KC977272	Bitzer et al. (2008), Kuhnert et al. (2014), Wendt et al. (2018)
*Rhopalostroma angolense*	CBS 126414	Ivory Coast		KY610420	KY610459	KY624228	KX271277	Wendt et al. (2018)
*Rosellinia aquila*	MUCL 51703	France		KY610392	KY610460	KY624285	KX271253	Wendt et al. (2018)
*Rosellinia buxi*	J.D.R. 99	France		GU300070	N/A	GQ844780	GQ470228	Hsieh et al. (2010)
*Rosellinia corticium*	MUCL 51693	France		KY610393	KY610461	KY624229	KX271254	Wendt et al. (2018)
*Rosellinia necatrix*	CBS 349.36	Argentina		AY909001	KF719204	KY624275	KY624310	Pelaez et al. (2008), Wendt et al. (2018)
*Rostrohypoxylon terebratum*	CBS 119137	Thailand	HT	DQ631943	DQ840069	DQ631954	DQ840097	Tang et al. (2007), Fournier et al. (2010)
*Ruwenzoria pseudoannulata*	MUCL 51394	D. R. Congo	HT	KY610406	KY610494	KY624286	KX271278	Wendt et al. (2018)
*Sarcoxylon compunctum*	CBS 359.61	South Africa		KT281903	KY610462	KY624230	KX271255	Senanayake et al. (2015), Wendt et al. (2018)
*Stilbohypoxylon elaeicola*	Y.M.J. 173	French Guiana		EF026148	N/A	GQ844826	EF025616	Hsieh et al. (2010)
*Stilbohypoxylon quisquiliarum*	Y.M.J. 172	French Guiana		EF026119	N/A	GQ853020	EF025605	Hsieh et al. (2010)
*Thamnomyces dendroidea*	CBS 123578	French Guiana	HT	FN428831	KY610467	KY624232	KY624313	Stadler et al. (2010), Wendt et al. (2018)
*Xylaria acuminatilongissima*	HAST 95060506	Taiwan	HT	EU178738	N/A	GQ853028	GQ502711	Hsieh et al. (2010)
*Xylaria adscendens*	J.D.R. 865	Thailand		GU322432	N/A	GQ844818	GQ487709	Hsieh et al. (2010)
*Xylaria arbuscula*	CBS 126415	Germany		KY610394	KY610463	KY624287	KX271257	Fournier et al. (2011), Wendt et al. (2018)
*Xylaria bambusicola*	WSP 205	Taiwan	HT	EF026123	N/A	GQ844802	AY951762	Hsieh et al. (2010)
*Xylaria brunneovinosa*	HAST 720	Martinique	HT	EU179862	N/A	GQ853023	GQ502706	Hsieh et al. (2010)
*Xylaria curta*	HAST 494	Martinique		GU322444	N/A	GQ844831	GQ495937	Hsieh et al. (2010)
*Xylaria discolor*	HAST 131023	USA	ET	JQ087405	N/A	JQ087411	JQ087414	Hsieh et al. (2010)
*Xylaria hypoxylon*	CBS 122620	Sweden	ET	KY610407	KY610495	KY624231	KX271279	Sir et al. (2016), Wendt et al. (2018)
*Xylaria multiplex*	HAST 580	Martinique		GU300098	N/A	GQ844814	GQ487705	Hsieh et al. (2010)
*Xylaria polymorpha*	MUCL 49884	France		KY610408	KY610464	KY624288	KX271280	Wendt et al. (2018)

^a^ATCC, American Type Culture Collection, Manassas, USA; BISH, Bishop Museum, Honolulu, USA; CBS, Westerdijk Fungal Biodiversity Institute, Utrecht, the Netherlands; GZU H, Guizhou University, Guiyang, China; HAST, Academia Sinica, Taipei, Taiwan; J.D.R., Jack D. Rogers, Washington State University, Pullman, USA; J.F., Jacques Fournier, Rimont, France; MFLUCC, Mae Fah Luang University, Chiang Rai, Thailand; MUCL, Université Catholique de Louvain, Louvain-la-Neuve, Belgium; STMA, Marc Stadler, Helmholtz-Zentrum für Infektionsforschung, Braunschweig, Germany; UCR, University of California, Riverside, USA; Y.M.J., Yu-Ming Ju, Academia Sinica, Taipei, Taiwan; WSP, Washington State University, Pullman, USA

^b^ET, epitype; HT, holotype; IT, isotype; PT, paratype

^c^N/A, not available; JGI, sequences retrieved from JGI-DOE (http://genome.jgi.doe.gov/)

### Morphology

Microscopic observations were made in tap water except where noted. Methods of microscopy included stereomicroscopy using a Nikon SMZ 1500 equipped with a Nikon DS-U2 digital camera, and Nomarski differential interference contrast (DIC) using a Zeiss Axio Imager.A1 compound microscope equipped with a Zeiss Axiocam 506 color digital camera. Images and data were gathered using the NIS-Elements D v. 3.22.15 or Zeiss ZEN Blue Edition software packages. Measurements are reported as maxima and minima in parentheses and the range representing the mean plus and minus the standard deviation of a number of measurements given in parentheses.

### Culture preparation, DNA extraction, PCR, and sequencing

Isolates were prepared from ascospores as described in Jaklitsch (2009) and grown on MEA or on 2% corn meal agar plus 2% *w*/*v* dextrose (CMD). Growth of liquid culture and extraction of genomic DNA was performed as reported previously (Voglmayr and Jaklitsch 2011; Jaklitsch et al. 2012) using the DNeasy Plant Mini Kit (QIAgen GmbH, Hilden, Germany).

The following loci were amplified and sequenced: the complete internal transcribed spacer region (ITS1–5.8S–ITS2) and a ca. 0.9-kb fragment of the large subunit nuclear ribosomal DNA (nuLSU rDNA), amplified and sequenced as a single fragment with primers V9G (de Hoog and Gerrits van den Ende 1998) and LR5 (Vilgalys and Hester 1990); a ca. 1.2-kb fragment of the RNA polymerase II subunit 2 (*RPB2*) gene with primers dRPB2-5f and dRPB2-7r (Voglmayr et al. 2016a); and a ca. 1.6-kb fragment of the beta-tubulin (*TUB2*) gene with primers T1D and T22D (Voglmayr et al. 2019). PCR products were purified using an enzymatic PCR cleanup (Werle et al. 1994) as described in Voglmayr and Jaklitsch (2008). DNA was cycle-sequenced using the ABI PRISM Big Dye Terminator Cycle Sequencing Ready Reaction Kit v. 3.1 (Applied Biosystems,Warrington, UK) and the PCR primers; in addition, primers ITS4 (White et al. 1990), LR2R-A (Voglmayr et al. 2012) and LR3 (Vilgalys & Hester 1990) were used as internal sequencing primers for the ITS-LSU rDNA region, and BtHV2r (Voglmayr et al. 2016b, 2017) and BtHVf (Voglmayr & Mehrabi 2018) for *TUB2*. Sequencing was performed on an automated DNA sequencer (ABI 3730xl Genetic Analyzer, Applied Biosystems).

### Data analysis

The newly generated sequences were aligned to the sequence alignments of Voglmayr et al. (2018), and GenBank sequences of four taxa of Diaporthales (*Gnomonia gnomon*, *Juglanconis juglandina*, *Linospora capreae*, and *Melanconis stilbostoma*) were added as the outgroup. Some taxa included in the matrix of Voglmayr et al. (2018) which contained poor or incomplete sequence data and which were not relevant for this study were removed from the matrices. The GenBank accession numbers of sequences used in these analyses are given in Table 1.

Sequence alignments for phylogenetic analyses were produced with the server version of MAFFT (http://mafft.cbrc.jp/alignment/server/), checked and refined using BioEdit v. 7.2.6 (Hall 1999). The ITS-LSU rDNA, *RPB2* and *TUB2* matrices were combined for subsequent phylogenetic analyses. After exclusion of ambiguously aligned regions and long gaps, the final combined data matrix contained 4718 characters (622 nucleotides of ITS, 1355 nucleotides of LSU, 1169 nucleotides of *RPB2* and 1572 nucleotides of *TUB2*). Familial classification of Xylariaceae and pylogenetically related families follows Voglmayr et al. (2018) and Wendt et al. (2018).

Maximum parsimony (MP) analyses were performed with PAUP v. 4.0a165 (Swofford 2002). All molecular characters were unordered and given equal weight; analyses were performed with gaps treated as missing data; the COLLAPSE command was set to MINBRLEN. MP analysis of the combined multilocus matrix was done using 1000 replicates of heuristic search with random addition of sequences and subsequent TBR branch swapping (MULTREES option in effect, steepest descent option not in effect). Bootstrap analyses with 1000 replicates were performed in the same way, but using 5 rounds of random sequence addition and subsequent branch swapping during each bootstrap replicate.

Maximum likelihood (ML) analyses were performed with RAxML (Stamatakis 2006) as implemented in raxmlGUI 1.3 (Silvestro and Michalak 2012), using the ML + rapid bootstrap setting and the GTRGAMMA substitution model with 1000 bootstrap replicates. The matrix was partitioned for the different gene regions. For evaluation and discussion of bootstrap support, values below 70% were considered low, between 70 and 90% medium/moderate and above 90% high.

## Results

### Molecular phylogeny

**Fig. 1:**
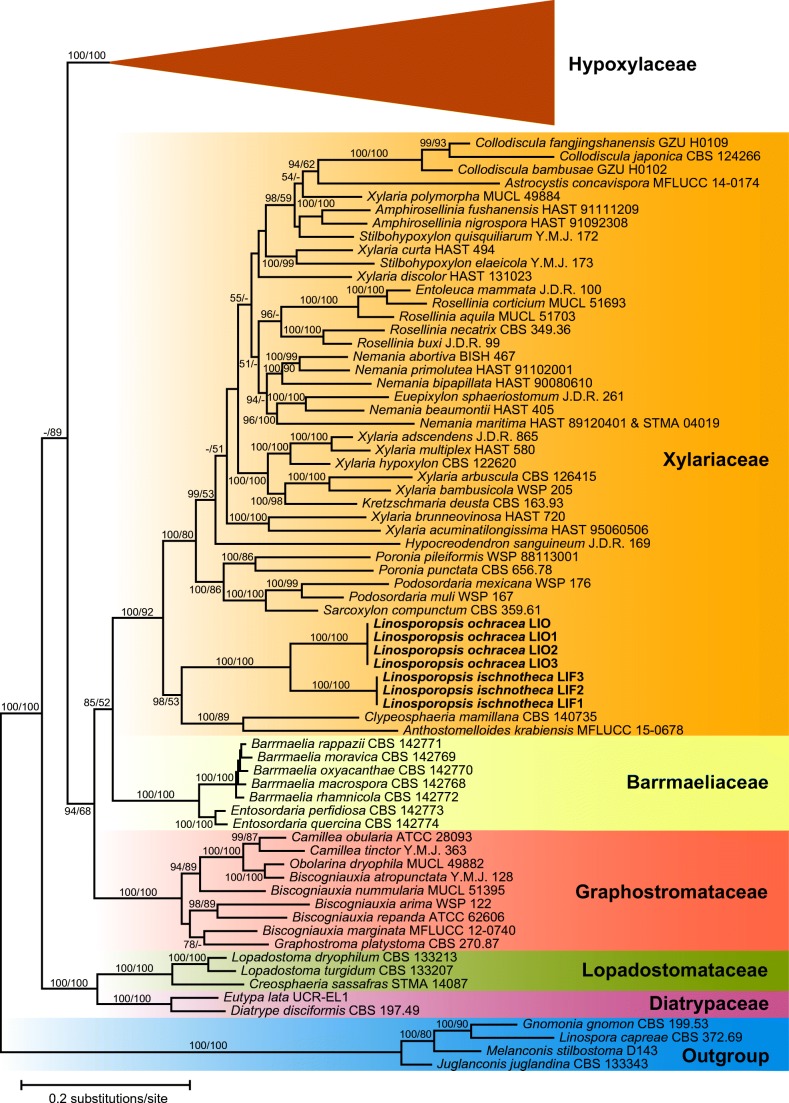
Simplified phylogram of the best ML trees (lnL = − 131,936.737) revealed by RAxML from an analysis of the combined ITS–LSU–*RPB2*–*TUB2* matrix of selected Xylariales, showing the position of *Linosporopsis* (bold). The large Hypoxylaceae clade, which is not treated in detail, is collapsed to provide sufficient space for the other clades of interest. ML and MP bootstrap support above 60% are given at the first and second positions, respectively, above or below the branches

The combined multilocus matrix used for phylogenetic analyses comprised 4718 characters, of which 2129 were parsimony informative (360 from ITS, 273 from LSU, 658 from *RPB2* and 838 from *TUB2*). Figure 1 shows a simplified phylogram of the best ML tree (lnL = − 131,936.737) obtained by RAxML. Maximum parsimony analyses revealed four MP trees 31,692 steps long, which were identical except for slightly different positions of *Daldinia andina* and *Stilbohypoxylon quisquiliarum* (not shown). The backbone of the MP trees was similar to the ML tree, except for a few minor topological differences of unsupported nodes within the Barrmaeliaceae, Graphostromataceae, Hypoxylaceae and Xylariaceae (not shown). *Linospora ischnotheca* and *L. ochracea* were revealed as closely related but distinct species with maximum support (Fig. 1). They were placed remotely from *Linospora capreae* (Diaporthales) in a basal position within Xylariaceae sensu stricto. A sister-group relationship with the highly (100%, ML) to moderately (89%, MP) supported *Clypeosphaeria mamillana*-*Anthostomelloides krabiensis* clade (Fig. 1) received high (98%, ML) or low (53%, MP) bootstrap support. The sequences of *Linospora ochracea* accessions from *Malus domestica*, *Pyrus communis*, and *Sorbus intermedia* were almost identical, confirming conspecificity of the accessions from these hosts.

## Taxonomy

***Linosporopsis*** Voglmayr & Beenken, gen. nov.

MycoBank: MB 833894.

*Etymology*: referring to its similarity to *Linospora*.

*Type species*: *Linosporopsis ischnotheca* (Desm.) Voglmayr & Beenken.

Mycelium in dead overwintered leaves, strongly bleaching the host tissue. Pseudostromata immersed in dead leaves, reduced, forming a distinct black clypeus-like structure on both sides of the leaf above and below the single perithecium, composed of dark brown, septate hyphae in dead host epidermis cells and forming a textura epidermoidea-intricata. Ascomata perithecial, scattered, solitary, immersed, (sub)globose, with a central apical papilla. Peridium thin, composed of hyaline, thin-walled, pseudoparenchymatous to prosenchymatous cells forming a textura angularis. Hamathecium of unbranched, thin-walled, hyaline, septate, apically tapering paraphyses. Asci unitunicate, long-cylindrical, with a short stipe, with an indistinct, inamyloid or slightly amyloid apical apparatus, containing 8 ascospores in a single fascicle. Ascospores long-filiform, hyaline, smooth, without visible septa, without sheath or appendages. Asexual morph unknown.

*Notes*: Within Xylariales, the genus is distinctive by long filiform ascospores without obvious septa and by single, scattered clypeate perithecia, which are embedded in a reduced pseudostroma immersed in dead, strongly bleached leaf tissue. The often large, bleached patches on the leaves are highly distinctive, especially when the leaves are wet. Unlike the large, amyloid, wedge-shaped apical apparatus of most Xylariaceae sensu stricto, that of *Linosporopsis* is indistinct and usually unnoticeable, and only occasionally slightly amyloid (observed only in a single accession each of *L. ochracea *and *L. magnagutiana*; see notes below).

**Fig. 2:**
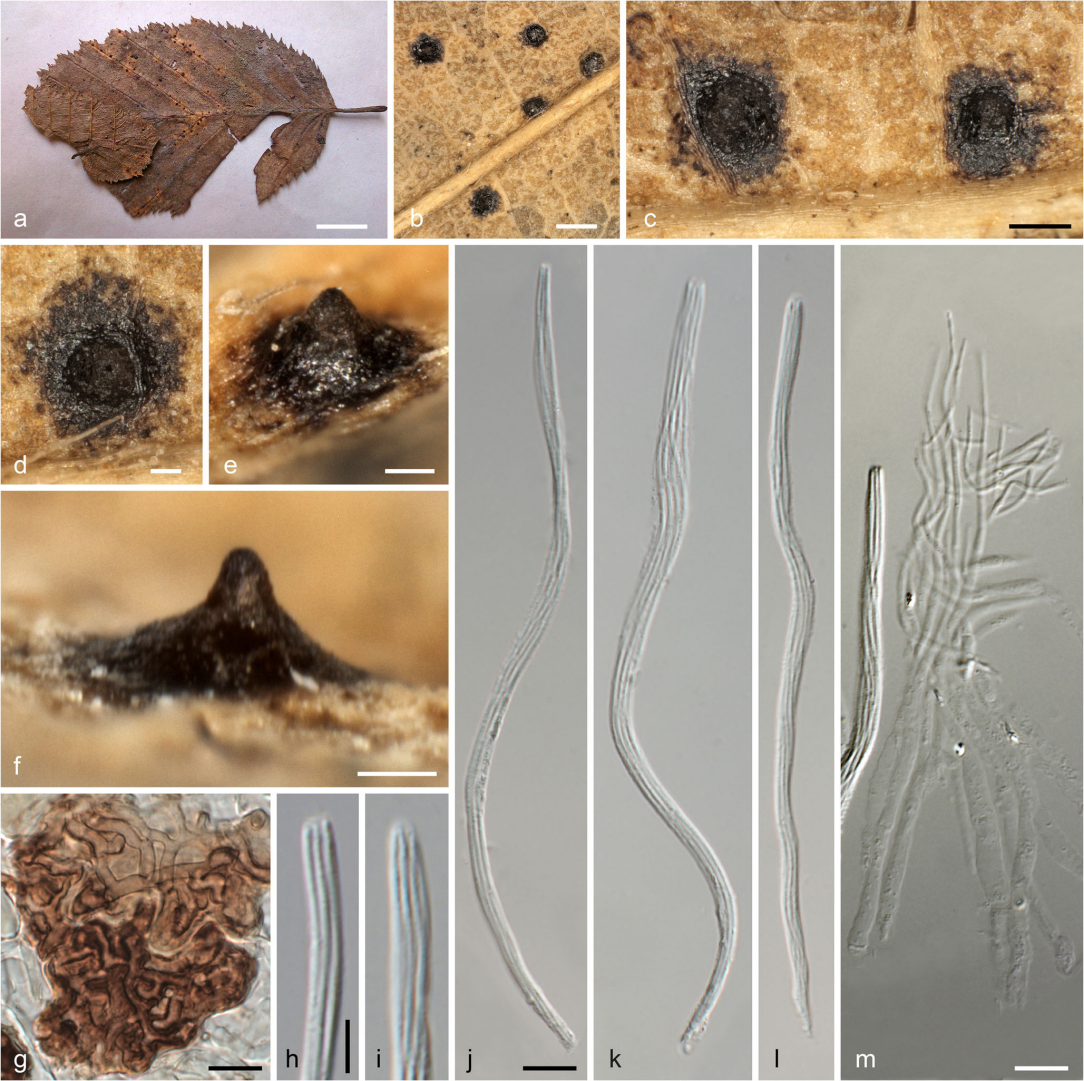
*Linosporopsis carpini* (W 2019-02783, isotype). **a** Colonies (bleached patches) on dead overwintered leaf of *Carpinus betulus*. **b** Close up of a colony with black clypeus-like uniperitheciate pseudostromata. **c**–**f** Uniperitheciate pseudostromata from above (**c**, **d**) and in side view (**e**, **f**). **g** Host epidermis cells with dark brown, septate, branched hyphae forming a textura epidermoidea-intricata. **h**, **i** Ascus apices. **j**–**l** Asci. **m** Paraphyses. All in 3% KOH. *Scale bars***a** 10 mm; **b** 400 μm; **c** 200 μm; **d**–**f** 100 μm; **g**, **j**–**m** 10 μm; **h**, **i** 5 μm

***Linosporopsis carpini*** (J. Schröt.) Voglmayr & Beenken, comb. nov. Fig. 2.

MycoBank: MB 833896.

*Basionym*. *Linospora carpini* J. Schröt., Hedwigia 15: 119. 1876.

Pseudostromata immersed in dead overwintered leaves, reduced, forming a distinct black clypeus (353–)384–463(–507) μm wide (*n* = 17) on both sides of the leaf, consisting of a textura epidermoidea-intricata composed of thick-walled, dark brown, septate hyphae 1.5–3 μm wide in dead host epidermis cells. Ascomata perithecial, scattered, solitary, immersed in dead leaf tissue, globose to ellipsoid, with a distinct central apical papilla 70–140(–185) μm wide at the base. Peridium not observed. Paraphyses unbranched, septate, thin-walled, collabent, 107–120 μm long, 3–5 μm wide at the base and gradually tapering to 1–1.2 μm at the tips. Asci (118–)135–160(–165) × (3.5–)3.7–4.5(–5.0) μm (*n* = 30), unitunicate, long-cylindrical, with a short stipe, with eight ascospores arranged in a single fascicle, with an indistinct inamyloid apical apparatus. Ascospores (120–)136–158(–162) × 0.7–1.1 μm, l/w = (110–)145–201(–230) (*n* = 30), filiform, with rounded ends, hyaline, without visible septa, without sheath or appendages.

No cultures available. No asexual morph observed.

*Habitat and host range*: Dead overwintered leaves of *Carpinus betulus*.

*Distribution*: Europe; only known from southwestern Germany and northern Italy.

*Isotypes*: Germany, Baden-Württemberg, Rastatt, Apr. 1876, J. Schröter, in Rabenhorst, Fungi Eur. Exs. 2132 (M-0304424, M-0304425, W 2019–02783).

*Notes*: Although no DNA data are yet available, morphology of ascomata, asci and ascospores leave no doubt that the species belongs to *Linosporopsis*, and considering the high host specificity of the genus, we recognize *L. carpini* as a distinct species. Apart from the type collection, this species is to our knowledge only known from an additional collection in northern Italy (Veneto, near Conegliano), which was collected in the same year as the type (Saccardo 1877). On the herbarium label of the type collection, it was stated to be common in the forests around Rastatt; however, we are not aware of any recent collections. The type collection has been edited and distributed in numerous copies in Rabenhorst, Fungi Eur. Exs. 2132, but we have investigated in detail only the copy deposited in W, that consists of a single leaf with a few perithecia. To save material, no sections were performed, and only a microscope preparation for documentation and measurements of asci, ascospores, paraphyses and clypeus hyphae was done. Our measurements revealed distinctly longer asci and ascospores than reported in the original description (118–165 μm vs. 70–80 μm in Rabenhorst 1876), which therefore is within the range of the other accepted *Linosporopsis* species.

**Fig. 3:**
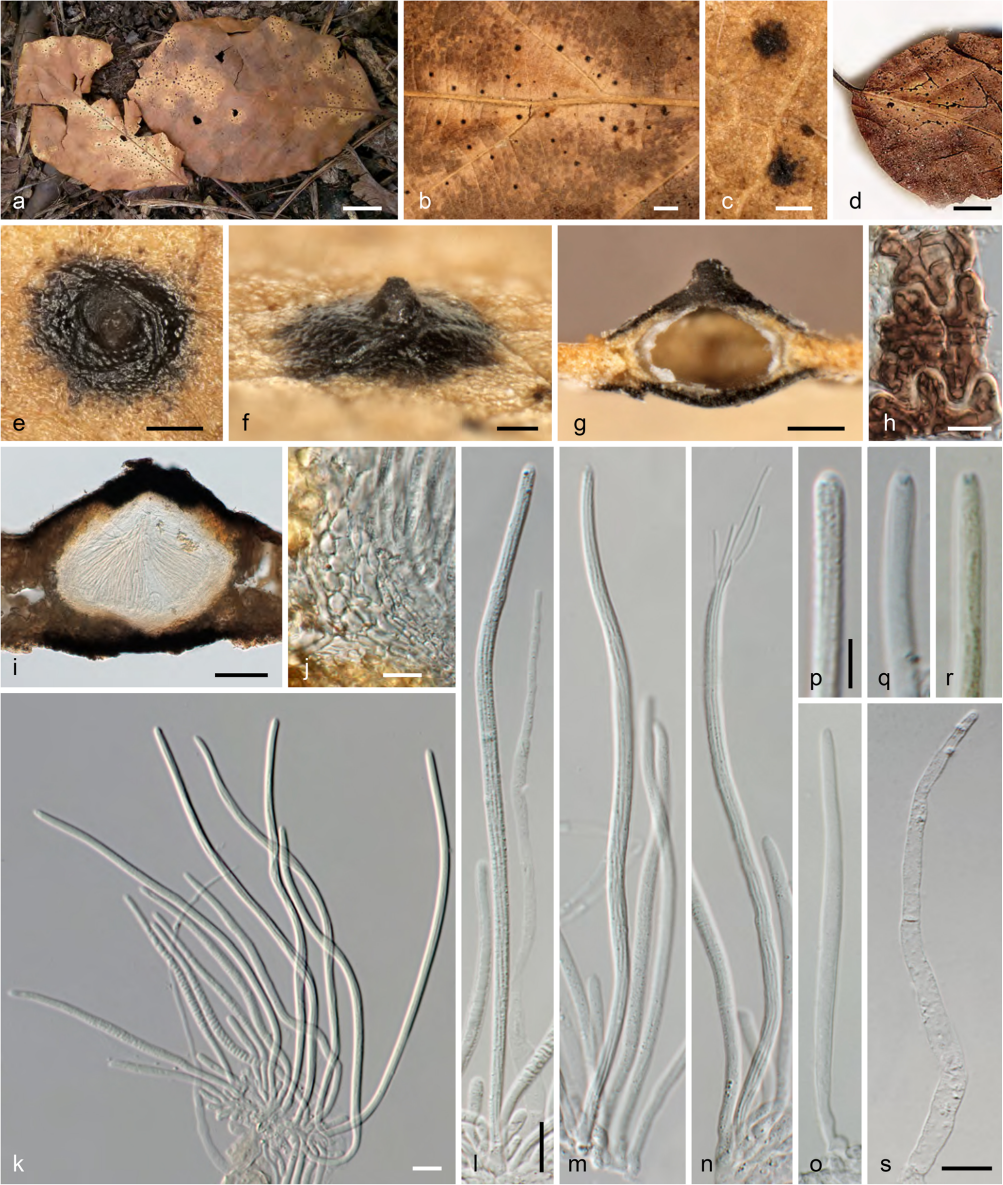
*Linosporopsis ischnotheca*. **a** Colonies (bleached patches) on dead overwintered leaves of *Fagus sylvatica* with scattered black, clypeus-like uniperitheciate pseudostromata. **b**–**d** Close up of colonies with black clypeus-like uniperitheciate pseudostromata. **e**–**g** Uniperitheciate pseudostromata from above (**e**), in side view (**f**), and in transverse section (**g**). **h** Host epidermis cells with dark brown, septate, branched hyphae forming a textura intricata. **i** Uniperitheciate pseudostroma in transverse section. **j** Pseudoparenchymatous, hyaline peridium and adjacent host tissue in section. **k**–**o** Asci (**o** immature). **p**–**r** Ascus apices. **s** Paraphysis. All in 3% KOH, except **i**, **j**, **p**, **s** in water; **r** in Lugol after KOH pre-treatment (**a**, **e**–**g**, **m**, **n** WU 40027; **b** PC0706583, isotype; **c** PC0706584, isotype; **d** PAD, holotype of *Linospora magnagutiana* subsp. *faginea*; **h** K(M) 206638, isotype; **i**, **j**, **p**, **s** WU 40026; **o**, **q**, **r** K(M) 206636, lectotype). *Scale bars***a**, **d** 10 mm; **b** 1 mm; **c**, **e** 200 μm; **f**, **g**, **i** 100 μm; **h**, **j**–**o**, **s** 10 μm, **p**–**r** 5 μm

***Linosporopsis ischnotheca*** (Desm.) Voglmayr & Beenken, comb. nov. Fig. 3.

MycoBank: MB 833895.

*Basionym*. *Sphaeria ischnotheca* Desm., Annls Sci. Nat., Bot., sér. 3 18: 365. 1852.

*Synonyms*. *Linospora faginea* Sacc., Michelia 1(no. 4): 405. 1878.

*Linospora ischnotheca* (Desm.) Sacc., Syll. fung. (Abellini) 2: 356. 1883.

Pseudostromata immersed in dead overwintered leaves, forming a distinct black clypeus (107–)145–247(–315) μm wide (*n* = 88) on both sides of the leaf, consisting of a textura epidermoidea-intricata composed of thick-walled, dark brown, septate hyphae 1.5–3 μm wide in dead host epidermis cells. Ascomata perithecial, scattered, solitary, immersed in dead leaf tissue, globose to ellipsoid, 230–340 μm diam., with a distinct central apical papilla 100–145(–160) μm wide at the base. Peridium (19–)22–32(–38) μm wide (*n* = 23), hyaline, pseudoparenchymatous, of hyaline isodiametric to elongate cells, marginal peridium cells (4.5–)7–13.5(–17) × (1.5–)2.5–4.5(–6.5) μm (*n* = 46), basal peridium cells smaller, (3–)4–9 (–10) × 1.5–2.3(–2.7) μm (*n* = 16). Paraphyses unbranched, septate, thin-walled, collabent, 74–110 μm long, 4.0–7.5 μm wide at the base and gradually tapering to 2–4.5 μm at the tips (*n* = 20). Asci (94–)122–153(–175) × (2.8–)3.4–4.3(–5.2) μm (*n* = 98), unitunicate, long-cylindrical, with a short stipe, with eight ascospores arranged in a single fascicle, with an indistinct inamyloid apical apparatus. Ascospores (84–)118–149(–170) × (0.6–)0.8–1.0(–1.3) μm, l/w = (35–)119–175(–205) (*n* = 55), filiform, with rounded ends, hyaline, without visible septa, without sheath or appendages.

Colonies on CMD and MEA white; aerial hyphae abundant. No asexual morph observed.

*Habitat and host range*: Dead overwintered leaves of *Fagus sylvatica* and *F. orientalis*; rarely also on *Quercus* sp.

*Distribution*: Europe; known from France, Germany, Italy, Spain, and Switzerland.

*Typification*: France, without place, date and collector, on dead leaves of *Fagus sylvatica*, in Desmazières, Pl. Crypt. N. France, Ed. 1, no. 2098 (K(M) 206636, lectotype of *Sphaeria ischnotheca* here designated, MBT 390204; PC 0706583, isotype); same collection, in Desmazières, Pl. Crypt. N. France, Ed. 1, no. 1798 (K(M) 206635, PC 0706584, isotypes). Italy, Veneto, Treviso, near Conegliano, spring 1877, C.L. Spegazzini (PAD, holotype of *Linospora faginea*). Switzerland, Zürich, Thuraue near Flaach, 13 May 2017, L. Beenken (WU 40024, epitype of *Sphaeria ischnotheca* here designated, MBT 390205; ex epitype culture CBS 145761 = LIF1).

*Other specimens examined*: France, Calvados (14), Caen, on dead leaves of *Fagus sylvatica*, without date, M.R. Roberge (M-0304427,? syntype). Landes, Lussagnet, 43.763725° N, − 0.223289° E, 140 m, 16 May 2017, A. Gross (ZT Myc 59965). Germany, Bavaria, Freising, Kranzberger Forst, Weltwald, on dead leaves of *Fagus orientalis*, 30 Apr. 2019, L. Beenken (WU 40033). Spain, Asturias, Gijón, on dead leaves of *Fagus sylvatica*, 16 Apr. 2015, Enrique Rubio Domínguez ERD 6431 (WU 40027). Ibid., on dead leaves of *Fagus sylvatica* and *Quercus robur*, 16 Apr. 2015, Enrique Rubio Domínguez (WU 40026; culture LIF3). Switzerland, Zürich, Ellikon am Rhein, 20 May 2017, L. Beenken (WU 40025, ZT Myc 59966; culture LIF2). Zürich, Winterthur, Eschenberg, 47°28′58″ N, 8°43′ 24″ E, 530 m, 16 May 2015, L. Beenken (ZT Myc 59967).

*Notes*: DNA sequence data and morphology place the species within Xylariaceae, as closest relative of *L. ochracea*. Desmazières (1851) first included specimens from leaves of *Fagus sylvatica* in his *Sphaeria ochracea*, but soon thereafter, he described them as a distinct species, *S. ischnotheca* (Desmazières 1852). In the protologue, he mentioned that the type collection contained only immature asci without spores, which was confirmed for all syntypes investigated in our study. The type collection was edited and distributed in two sets as Pl. Crypt. N. France, Ed. 1, nos. 1798 and 2098, which is also mentioned in the protologue. Neither locality nor collector are mentioned on the herbarium labels and in the original description of the species, and no original notes of Desmazières are attached to the two copies present in PC. However, the herbarium labels of a specimen in M, probably also a syntype, indicates that it was collected by M.R. Roberge in Caen, i.e. the same place and collector as the type of *L. ochracea* (see below), which appears plausible considering that material of *Fagus* was mentioned in the original description of *L. ochracea*. As the type collection of *Sphaeria ischnotheca* is immature, we here designate a recent mature collection, for which a culture and DNA sequences are available, as epitype to stabilize the species nomenclature.

*Linospora faginea*, which was also described from dead leaves of *Fagus sylvatica*, is obviously a synonym of *L. ischnotheca*; the protologue in Saccardo (1878) fully matches our material. As Saccardo material of PAD is not sent out on loan, we have not been able to investigate the type in detail, but the illustrations of the specimen and label kindly provided by the Erbario dell’Università di Padua show that it agrees with *L. ischnotheca* (see Fig. 3d).

The inamyloid apical apparatus of *L. ischnotheca* is usually indistinct, and only well-seen in IKI (Fig. 3r) or cotton blue. For beautiful additional illustrations of the Spanish specimen ERD 6431, see also http://www.ascofrance.com/search_forum/35346.

**Fig. 4:**
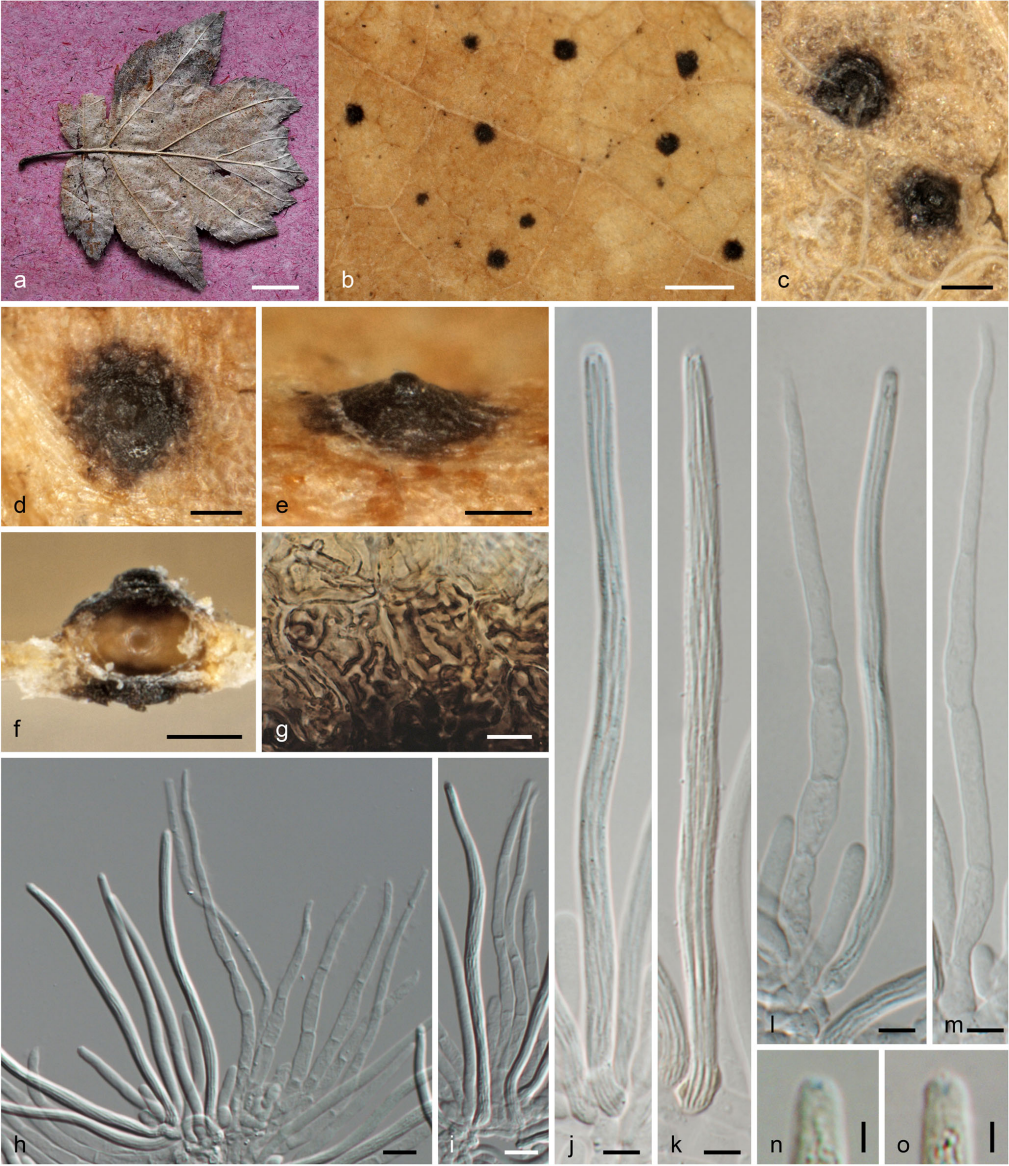
*Linosporopsis magnagutiana*. **a** Colonies on dead overwintered bleached leaf of *Sorbus torminalis*. **b** Close up of a colony with black clypeus-like uniperitheciate pseudostromata. **c**–**f** Uniperitheciate pseudostromata from above (**c**, **d**), in side view (**e**) and in transverse section (**f**). **g** Host epidermis cells with dark brown, septate, branched hyphae forming a textura epidermoidea-intricata. **h–l﻿** Asci with paraphyses (**h**, **j**, **l**). **m** Paraphysis. **n**, **o** Ascus apices with slightly amyloid ring. All in 3% KOH, except **k**, **n**, **o** Lugol after KOH pre-treatment (**a**–**m** Thümen, Mycoth. Univ. 1454 (**a** M s.n., **b**–**m** WU s.n.); **n**, **o** Saccardo, Mycoth. Ven. 1352 (WU s.n.)). *Scale bars***a** 10 mm; **b** 1 mm; **c**–**f** 100 μm; **g**–**i** 10 μm; **j**–**m** 5 μm; **n**, **o** 2 μm

***Linosporopsis magnagutiana*** (Sacc.) Voglmayr & Beenken, comb. nov. Fig. 4.

MycoBank: MB 833897.

*Basionym*. *Linospora magnagutiana* Sacc., Michelia 1(no. 1): 45. 1877.

Pseudostromata immersed in dead overwintered leaves, reduced, forming a distinct black clypeus (109–)126–203(–294) μm wide (*n* = 42) on both sides of the leaf, consisting of a textura epidermoidea-intricata composed of thick-walled, dark brown, septate hyphae 2–4 μm wide mostly in dead host epidermis cells. Ascomata perithecial, scattered, solitary, immersed in dead leaf tissue, globose to depressed globose, ca. 150–170 μm diam., with a distinct central apical papilla 30–65 μm wide at the base. Paraphyses unbranched, septate, thin-walled, collabent, (73–)81–100(–111) μm long, (3.5–)4–5.5(–6) μm wide at the base and gradually tapering to (1.2–)1.6–2.3(–2.6) μm at the tips (*n* = 23). Asci (79–)94–121(–137) × (3.5–)4.2–5.3(–6.2) μm (*n* = 96), unitunicate, long-cylindrical, with a short stipe, with eight ascospores arranged in a single fascicle, with an indistinct inamyloid to slightly amyloid apical apparatus. Ascospores (73–)90–116(–132) × (0.7–)0.8–1(–1.3), l/w = (74–)94–137(–174) (*n* = 89), with rounded ends, hyaline, without visible septa, without sheath or appendages.

No cultures available. No asexual morph observed.

*Habitat and host range*: Dead overwintered leaves of *Sorbus torminalis*.

*Distribution*: Europe; only known from northern Italy.

*Holotype*: Italy, Veneto, Mantova, Bosco della Fontana, on dead leaves of *Sorbus torminalis*, Apr. 1873, A. Magnaguti-Rondini (PAD, not seen).

*Specimens examined*: Italy, Veneto, Conegliano, on dead leaves of *Sorbus torminalis*, summer 1878, C. Spegazzini, in Saccardo, Mycoth. Ven. 1352 (WU s.n.). Same place, May 1878, C. Spegazzini, in Baglietto, Cesati & Notaris, Erb. Critt. Ital. Ser. II 727 (M-0304429, Z Myc 8040). Same place, Apr. 1879, C. Spegazzini, in Thümen, Mycoth. Univ. 1454 (M-0304428, WU s.n., ZT Myc 60357).

*Notes*: Due to the lack of fresh specimens, no cultures and sequence data are available for *L. magnagutiana*, but its morphology clearly places it in *Linosporopsis*. Only few historic records from northern Italy, all collected in the 1870ies, are known. We have not been able to investigate the type from PAD, which is not sent out on loan, but two additional authentic collections from the same area were available for study. As the historic material is very brittle, no useable section of the peridium could be prepared. The rosaceous host, *Sorbus torminalis*, and similar morphology indicates that *L. magnagutiana* may be conspecific with *L. ochracea*. However, in one locality (Bayerisches Landesarboretum “Weltwald”), where leaves of *Pyrus domestica* and *Sorbus latifolia* were heavily infected by *L. ochracea*, no *Linosporopsis* could be found on leaves of directly close-by *Sorbus torminalis*, indicating that they are distinct. In addition, the asci and ascospores of *L. magnagutiana* are slightly shorter than those of *L. ochracea* ((79–)94–121(–137) and (73–)90–116(–132) μm vs. (91–)108–130(–153) and (88–)103–126(–149) (*n* = 139) μm, respectively), and also its clypei are somewhat smaller ((109–)126–203(–294) vs. (97–)172–276(–355) μm). Therefore, for the time being, we argue for maintaining them as distinct species.

**Fig. 5:**
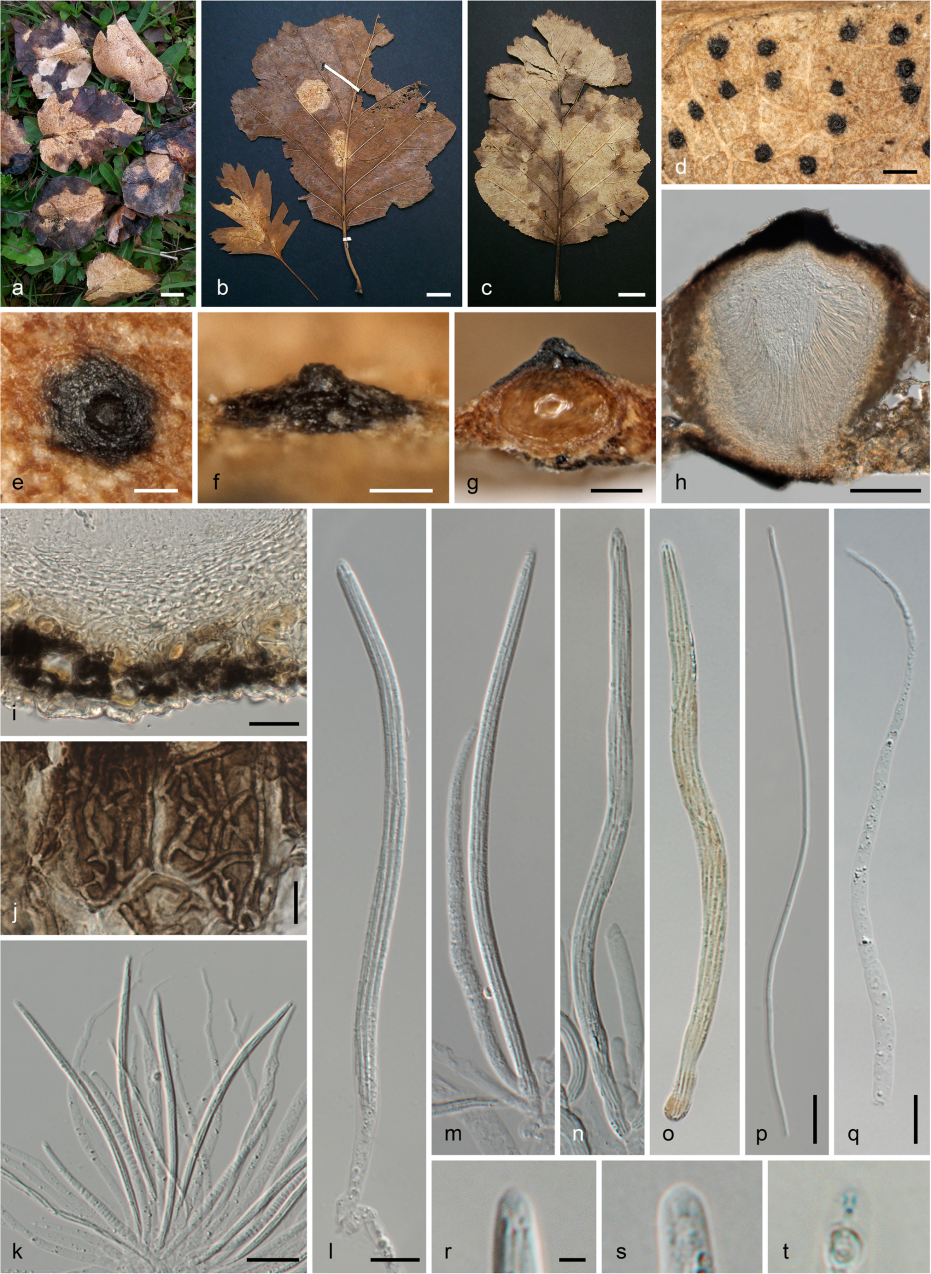
*Linosporopsis ochracea*. **a**–**c** Colonies (bleached patches) on dead overwintered leaves of *Pyrus communis* (**a**), *Crataegus* sp. (left) and *Sorbus latifolia* (right) (**b**), and *Sorbus intermedia* (**c**), with scattered black, clypeus-like uniperitheciate pseudostromata. **d** Close up of colony with black clypeus-like uniperitheciate pseudostromata. **e**–**g** Uniperitheciate pseudostromata from above (**e**), in side view (**f**), and in transverse section (**g**). **h** Uniperitheciate pseudostroma in transverse section. **i** Pseudoparenchymatous, hyaline peridium, adjacent host tissue and lower clypeus in section. **j** Host epidermis cells with dark brown branched hyphae. **k**–**o** Asci. **p** Ascospore. **q** Paraphysis. **r**–**t** Ascus apices. All in 3% KOH, except **h**, **l**, **p**, **q** in water; **o**, **t** in Lugol after KOH pre-treatment (**a**, **d**, **g**–**j**, **l**, **p** WU 40029; **b**, **f**, **n**, **o**, **r** PC 0706581, lectotype; **c**, **e**, **k**, **m**, **q** WU 40031, epitype; **s** PC 0706581; **t** WU 40028. *Scale bars***a**–**c** 10 mm; **d** 500 μm; **e**–**h** 100 μm; **i**, **k** 20 μm; **j**, **l**–**q** 10 μm; **r**–**t** 2 μm

***Linosporopsis ochracea*** (Sacc.) Voglmayr & Beenken, comb. nov. Fig. 5.

MycoBank: MB 833898.

*Basionym*. *Linospora ochracea* Sacc., Syll. fung. (Abellini) 2: 355. 1883.

*Replaced synonym*. *Sphaeria ochracea* Desm., Annls Sci. Nat., Bot., sér. 3 16: 317. 1851, nom. illegit. Art. 53.1, non *Sphaeria ochracea* Pers., Syn. meth. fung. (Göttingen) 1: 18. 1801.

Pseudostromata immersed in dead overwintered leaves, reduced, forming a distinct black clypeus (97–)172–276(–355) μm wide (*n* = 143) on both sides of the leaf, consisting of a textura epidermoidea-intricata composed of thick-walled, dark brown, septate hyphae 1.5–3.7 μm wide mostly in dead host epidermis cells. Ascomata perithecial, scattered, solitary, immersed in dead leaf tissue, globose to depressed globose, 180–260 μm diam., with a distinct central apical papilla (45–)60–89(–114) μm wide at the base (*n* = 88). Peridium (22–)26–37(–41) μm wide (*n* = 20), hyaline, pseudoparenchymatous, of hyaline isodiametric to elongate cells, marginal peridium cells (6.2–)8.5–14.8(–17.3) × (3.7–)4.8–7.7(–10) μm (*n* = 25), basal peridium cells smaller, (4–)5–9.5(–11.3) × (1.7–)2.5–4.2(–5) μm (*n* = 26). Paraphyses unbranched, septate, thin-walled, collabent, 75–160 μm long, 3–6(–9.7) μm wide at the base and gradually tapering to 1–2 μm at the tips (*n* = 34). Asci (91–)108–130(−153) × (3–)4–5.5(–6.7) μm (*n* = 205), unitunicate, long-cylindrical, with a short stipe, with eight ascospores arranged in a single fascicle, with an indistinct inamyloid or slightly amyloid apical apparatus. Ascospores (88–)103–126(−149) × (0.8–)0.9–1.3(–1.6) μm, l/w = (62–)87–132(–174) (*n* = 139), filiform, with rounded ends, hyaline, without visible septa, without sheath or appendages.

Colonies on CMD and MEA white; aerial hyphae abundant. No asexual morph observed.

*Habitat and host range*: Dead overwintered leaves of various Rosaceae, subtribus Pyrinae; e.g., *Crataegus* spp., *Cydonia oblonga*, *Malus domestica*, *Mespilus germanica*, *Pyrus* spp. and *Sorbus* spp.

*Distribution*: Europe; known from Austria, France, Germany, Italy and Switzerland.

*Typification*: France, Calvados (14), Caen, Hérouville-Saint-Clair, Parc de Lébisey, on dead leaves of *Crataegus monogyna* and *Sorbus latifolia*, May 1850, M.R. Roberge, in Desmazières, Pl. Crypt. N. France, Ed. 1, no. 2099 (PC 0706581, lectotype of *Linospora ochracea* here designated, MBT 390206; K(M) 206803, K(M) 206804, K(M) 206805, K(M) 206,806, PC 0706579, isotypes). Germany, Bavaria, Freising, Kranzberger Forst, Bayerisches Landesarboretum “Weltwald”, on dead leaves of *Sorbus intermedia*, 30 Apr. 2019, L. Beenken (WU 40031, epitype of *Linospora ochracea* here designated, MBT 390207, isoepitype ZT Myc 59968; ex epitype culture CBS 145999 = LIO3).

*Other specimens examined*: Austria, Niederösterreich, Marchegg, at the railroad embankment near the river March, on dead leaf of *Malus domestica*, 1 May 2019, H. Voglmayr (WU 40032); Oberösterreich, Raab, Wetzlbach, on dead leaves of *Pyrus communis*, 23 Mar. 2019, H. Voglmayr (WU 40029; culture LIO1). France, Calvados (14), Caen, Hérouville-Saint-Clair, Parc de Lébisey, on dead leaves of *Pyrus argentea*, Apr. 1851, M.R. Roberge (K(M) 206645, PC 0706580); same collection data, in Desmazières, Pl. Crypt. N. France, Ed. 1, no. 2100 (K(M) 206641, K(M) 206642, K(M) 206644, PC 0706582); same collection data, in Desmazières, Pl. Crypt. N. France, Ed. 2, Ser. 1, no. 1800 (K(M) 206643); same place, collector and host, without date (M-0304431); same place and collector, on dead leaves of *Sorbus* sp., without date (M-0304430). Germany, Bavaria, Freising, Kranzberger Forst, Weltwald, on dead leaves of *Pyrus communis*, 30 Apr. 2019, L. Beenken (WU 40030, ZT Myc 59969; culture LIO2). Switzerland, Zürich, Henggart, on dead leaves of *Malus domestica*, 13 May 2017, L. Beenken (WU 40028, ZT Myc 59970; culture CBS 145760 = LIO).

*Notes*: DNA sequence data and morphology place the species within Xylariaceae, as closest relative of *L. ischnotheca*. It was first described as *Sphaeria ochracea* by Desmazières (1851), but the name is illegitimate as it is a younger homonym of *Sphaeria ochracea* Pers. (1801). Therefore, *Linospora ochracea* Sacc., originally established as a new combination of *Sphaeria ochracea* Desm., is to be treated as a replacement name and represents the valid basionym.

In the protologue, Desmazières (1851) listed leaves of *Crataegus*, *Cydonia*, *Mespilus*, *Sorbus* and also *Fagus* as hosts; however, no collection or specimen data were given. For the specimens on *Fagus*, Desmazières (1852) subsequently described a distinct species, *Sphaeria ischnotheca* (see above). As concluded from the original material of Desmazières in PC and K, and from his notes attached to the specimen PC 0706581, the species was based on material collected by M.R. Roberge in Hérouville-Saint-Clair near Caen in May 1850, which Desmazières edited in his Pl. Crypt. N. France, Ed. 1, no. 2099. This exsiccatum contains material from *Crataegus monogyna* and *Sorbus latifolia*. From the same locality, Desmazières also distributed material from *Pyrus argentea* (as Pl. Crypt. N. France, Ed. 1, no. 2100 and Pl. Crypt. N. France, Ed. 2, Ser. 1, no. 1800), under the unpublished name *Sphaeria ochracea* f. *pyrina*, which, however, does not qualify for the type, as this host is not listed in the protologue; in addition, it was collected one year later (Apr. 1851) than the type, which may be a reason why this host was not cited in the protologue.

Unlike all other accessions of *L. ochracea* investigated by us, which had an indistinct, inamyloid apical apparatus, the Swiss collection WU 40028 from *Malus domestica* showed a tiny, wedge-shaped, slightly amyloid apical apparatus after KOH pre-treatment (see Fig. 5t). However, the sequences obtained from this accession fully matched the other collections, indicating a variable iodine reaction that probably depends on the maturity and preservation of the specimen.

**Key to the species of** ***Linosporopsis***

- 1. On leaves of Rosaceae..................................................2
- 1. On leaves of Fagaceae (*Fagus*, *Quercus*) or Betulaceae (*Carpinus*).........................................................................3
- 2. On leaves of *Sorbus torminalis............L. magnagutiana*
- 2. On leaves of other rosaceous hosts (*Crataegus*, *Cydonia*, *Malus*, *Pyrus*, *Sorbus*)......................*L. ochracea*
- 3. On leaves of *Fagus*; occasionally also *Quercus .....................................................................L. ischnotheca*
- 3. On leaves of *Carpinus ....................................L. carpini*

## Discussion

The results of our molecular phylogenetic investigations confirmed the conclusions of Monod (1983) that the species treated here are not congeneric with *Linospora* and do not belong to Diaporthales. However, while he assumed that they belong to *Ophiodothella*, currently classified within Phyllachoraceae (Phyllachorales), our phylogenetic analysis placed them in a basal clade of Xylariaceae sensu stricto (Xylariales). Based on the presence of an amyloid apical ascus ring, conidia resembling Diatrypaceae and a single nuSSU rNDA sequence, Hanlin et al. (2002) assumed xylarialean affinities of *Ophiodothella*; however, these conclusions were based on non-type species and need to be verified by re-investigation of the generic type. No type material of the generic type, *O. atromaculans* (Henn.) Höhn., is extant in B where the material of Hennings is kept (R. Lücking, personal communication). However, even if xylarialean, the following features do not support that *Ophiodothella* is congeneric with the species treated here: an obligate parasitic lifestyle in living leaves, a tropical to subtropical distribution almost exclusively in the New World, formation of pycnidial or acervular conidiomata, lack of distinct bleaching of the substrate and morphological differences of the ascomata (Hanlin et al. 1992, 2002, 2018). Particularly the generic type, *O. atromaculans*, deviates significantly from our species by an extended effuse, black stromatic crust (Hennings 1904; Hanlin et al. 1992). Additional genera with solitary clypeate ascomata and filiform ascospores that were previously attributed to Xylariales include *Linocarpon* and *Neolinocarpon*; however, these have been shown to belong to Chaetosphaeriales by sequence data (Konta et al. 2017). As no suitable described genus is available within Xylariaceae, we establish the new genus *Linosporopsis* for them.

Sister group relationship of *Linosporopsis* to the *Clypeosphaeria mamillana*-*Anthostomelloides krabiensis* clade is highly supported in the ML analyses, but receives only low support in the MP analyses. *Linosporopsis* is similar to the latter species in solitary ascomata of similar size that are embedded in a reduced pseudostroma within the host tissue and shares a distinct clypeus and apical papilla with *Clypeosphaeria mamillana*. However, marked differences to *Linosporopsis* include ellipsoid to oblong brown ascospores; a large, wedge-shaped, strongly amyloid apical ascus apex; and, in *A. krabiensis*, the lack of a clypeus and of an apical papilla (Jaklitsch et al. 2016; Tibpromma et al. 2017).

Ecologically, there is evidence that *Linosporopsis* occupies a niche as a leaf endophyte, and there is so far no indication of parasitism. Observations in Austrian and Swiss sites with abundant sporulation of *Linosporopsis ochracea* on dead overwintered *Pyrus* and *Malus* leaves revealed no obvious symptoms on living *Pyrus* and *Malus* leaves during the following summer. Evidently, the life cycle of *Linosporopsis* is connected with that of their hosts, as the short-lived ascospores are only produced briefly after their hosts unfold their new leaves in spring. These young leaves are then infected by the ascospores to complete the life cycle, with the living leaf tissue remaining asymptomatic during the growing season. After leaf abscission, the mycelium continues growth on the fallen leaves during the winter season, causing a distinctive bleaching of the decaying leaves, and finally ascomata and ascospores are produced again in the following spring.

The filiform, hyaline ascospores of *Linosporopsis* are very unusual for Xylariaceae, which mostly have more or less ellipsoid, brown ascospores, and therefore, the placement of *Linosporopsis* within Xylariaceae sensu stricto is somewhat surprising. However, ascospore morphology has proven not to be a good character for family segregation in the Xylariales, while the asexual morphs seem to agree better with the phylogeny (Ju and Rogers 1996, 2002; Wendt et al. 2018). So far, no asexual morph is known for *Linosporopsis*. The hyaline, filiform spores are likely an adaptation to colonization and infection of living leaves of trees. While little understood and investigated in detail, there is strong evidence that long, curved spores are effective adaptations to facilitate attachment on vertical or otherwise challenging exposed surfaces and are therefore advantageous for successful germination and establishment on aerial plant parts (Calhim et al. 2018). It is therefore not surprising that filiform ascospores have independently evolved in leaf-inhabiting species of various ascomycete lineages. This also provides an explanation for the morphological similarities to the unrelated diaporthalean genus *Linospora*, which has a similar ecology.
